# Automated Generation of Radiologic Descriptions on Brain Volume Changes From T1-Weighted MR Images: Initial Assessment of Feasibility

**DOI:** 10.3389/fneur.2019.00007

**Published:** 2019-01-24

**Authors:** Kentaro Akazawa, Ryo Sakamoto, Satoshi Nakajima, Dan Wu, Yue Li, Kenichi Oishi, Andreia V. Faria, Kei Yamada, Kaori Togashi, Constantine G. Lyketsos, Michael I. Miller, Susumu Mori

**Affiliations:** ^1^Department of Radiology, Johns Hopkins University School of Medicine Baltimore, MD, United States; ^2^Department of Radiology, Graduate School of Medical Science, Kyoto Prefectural University of Medicine Kyoto, Japan; ^3^Department of Diagnostic Imaging and Nuclear Medicine, Kyoto University Graduate School of Medicine Kyoto, Japan; ^4^AnatomyWorks, LLC Baltimore, MD, United States; ^5^Division of Geriatric Psychiatry and Neuropsychiatry, Memory and Alzheimer's Treatment Center & Alzheimer's Disease Research Center, Department of Psychiatry and Behavioral Sciences, Johns Hopkins University School of Medicine Baltimore, MD, United States; ^6^Department of Psychiatry and Behavioral Sciences, Johns Hopkins University Baltimore, MD, United States; ^7^Department of Biomedical Engineering, Johns Hopkins University Baltimore, MD, United States

**Keywords:** automated generation, radiologic description, dementia, brain atrophy, 3D T1 weighted image, brain atlas

## Abstract

**Purpose:** To examine the feasibility and potential difficulties of automatically generating radiologic reports (RRs) to articulate the clinically important features of brain magnetic resonance (MR) images.

**Materials and Methods:** We focused on examining brain atrophy by using magnetization-prepared rapid gradient-echo (MPRAGE) images. The technology was based on multi-atlas whole-brain segmentation that identified 283 structures, from which larger superstructures were created to represent the anatomic units most frequently used in RRs. Through two layers of data-reduction filters, based on anatomic and clinical knowledge, raw images (~10 MB) were converted to a few kilobytes of human-readable sentences. The tool was applied to images from 92 patients with memory problems, and the results were compared to RRs independently produced by three experienced radiologists. The mechanisms of disagreement were investigated to understand where machine–human interface succeeded or failed.

**Results:** The automatically generated sentences had low sensitivity (mean: 24.5%) and precision (mean: 24.9%) values; these were significantly lower than the inter-rater sensitivity (mean: 32.7%) and precision (mean: 32.2%) of the radiologists. The causes of disagreement were divided into six error categories: mismatch of anatomic definitions (7.2 ± 9.3%), data-reduction errors (11.4 ± 3.9%), translator errors (3.1 ± 3.1%), difference in the spatial extent of used anatomic terms (8.3 ± 6.7%), segmentation quality (9.8 ± 2.0%), and threshold for sentence-triggering (60.2 ± 16.3%).

**Conclusion:** These error mechanisms raise interesting questions about the potential of automated report generation and the quality of image reading by humans. The most significant discrepancy between the human and automatically generated RRs was caused by the sentence-triggering threshold (the degree of abnormality), which was fixed to z-score >2.0 for the automated generation, while the thresholds by radiologists varied among different anatomical structures.

## Introduction

Radiologic assessments of brain magnetic resonance (MR) images are solely based on subjective judgment utilizing radiologists' knowledge and experience. The input of the process is a set of MR images with different contrasts, and the output is free-text. The content of the text (hereafter known as radiologic report [RR]) is typically a description of remarkable anatomic findings and often, but not always, includes a diagnosis. In this process, we can consider the role of the radiologist as translating the anatomic features captured in the MR images to clinically meaningful language (i.e., semantic labels). During this translation, features judged to be within the normal range are filtered out, while abnormalities that are visually appreciable and judged as clinically important are documented. One of the most important aspects of this process is that it reduces three-dimensional MR images of ~10 MB in size into a few kilobytes of clinically meaningful and human-understandable information, which can be shared with other physicians and patients. To perform this conversion of high-dimensional imagery to semantic labels, the ability of humans is generally understood to be far superior to that of computer algorithms. However, the conversion by humans is criticized for its accuracy, efficiency, and consistency (1–4). In the era of modern medical informatics and big data analytics, the thought process behind this huge data contraction from 10 MB to few KB is not documented and thus not available in a readily usable format for retroactive large-scale analyses.

In the past two decades, technologies for quantitative analyses of brain MRI, especially voxel-based analysis and automated segmentation, have advanced considerably. Given the known shortcomings of the subjective image reading, we may wonder why these advanced quantitative tools have been rarely adopted for clinical image reading. There seem to be important gaps between the numbers provided by the tools and the actual requirements of radiologists. Suppose the RRs in a free-text format are the final outcome of brain MRI reading (which could be a simplification), one of the approaches to close the gap would be to directly generate RRs by computer algorithms.

Although we do not yet know if human-readable outputs would be the best solution for future computer-assisted diagnosis of MR images, we expected such efforts would deepen our understanding about the mechanism of the image reading by humans. In reality, radiological reading is a complicated process, because radiologists read multiple images (e.g., T1-weighted, T2-weighted, and fluid-attenuated images), evaluate both anatomy (sizes and shapes) and image intensity profiles, and integrate clinical information (e.g., the reason for MRI scans) to provide an RR. In this study, we inevitably needed to simplify the paradigm. The simplifications we adopted were as follows:

➢ We focused on patients who visited our memory clinics (excluding stroke and tumor cases). It is known that MRI has limited value for diagnosis or patient care of subjects with memory problems. However, in routine clinical practice, most of this patient population receives MRI scans, and radiologists provide their radiological reports. This patient population has both normal reports and cases with noticeable abnormalities, most frequently accelerated brain atrophy and thus, often leads to abnormality-positive radiological reports. This relatively large effect size is preferable for the purpose of this study, compared to populations dominated by negative findings.
➢ We focused on anatomical reading of T1-weighted images, specifically magnetization-prepared rapid gradient-echo (MPRAGE) scans. Radiological reading based on T1-weighted images tends to be focused on anatomy (sizes and shapes) of brain structures. This is technically more straightforward than evaluating abnormalities in signal intensities in multiple contrasts.

On the basis of these simplified radiological domains, we developed a tool that automatically translates MR images into RRs. In the past, there were various efforts to create structured reports at the time of image reading or to develop tools to systematically analyze existing free-text reports retrospectively (5–9). However, to our best knowledge, this is one of the first attempts to generate human-readable RRs directly from raw MR images. In this study, we used a state-of-the-art multi-atlas brain segmentation tool (10–19) and developed an interpreter that connects the anatomical quantification results to the RRs. To test the accuracy of the sentence generator, reports from three radiologists were compared with automated reports generated for 92 patients. Our primary goal was to measure the accuracy of the computer-generated sentences. However, during the research, we encountered many interesting results and challenges. These findings are of fundamental importance in understanding radiologists' thought processes and for the future development of smarter algorithms and large-scale evidence-based studies. Herein, we aim to present our study results, particularly with respect to the mechanisms of disagreement between radiologists and the automated analyses.

## Materials and Methods

### Data Sources: Normal Data

Approval was obtained from the Johns Hopkins Medicine Institutional Review Board to access and use the MRI reports. To develop a tool that can be applied to clinical practices, the tool cannot rely on data that need to follow stringent criteria (i.e., data need to be acquired by the same scanner with a fixed protocol). To ensure that the normal data had the expected range of variability, we included data from various sources with a reasonable degree of difference in their imaging protocols (20). However, all sources conformed to the definition of MPRAGE-type scans with <1.5-mm resolution. The data sources included the following:

1. Alzheimer's Disease Neuroimaging Initiative (ADNI) sagittal data (RRID:SCR_003007): These data were obtained from the ADNI database (adni.loni.usc.edu). The ADNI was launched in 2003 as a public–private partnership, led by Principal Investigator Michael W. Weiner, MD. Its primary goal is to assess whether serial MRI, positron emission tomography, other biological markers, or clinical and neuropsychological assessments can be combined to measure the progression of mild cognitive impairment and early Alzheimer's disease.
  We used data on cognitive normal controls (*n* = 72) from the ADNI database obtained using six different types of MRI hardware with both 1.5T and 3.0T MRI (GE Healthcare [Waukesha, WI]; Siemens Healthcare [Erlangen, Germany]; and Philips Medical Systems [Best, The Netherlands]). The mean (±SD) age of the individuals was 74.1 ± 6.4 years (age range: 56–96 years).
2. Johns Hopkins University internal 3T sagittal data: These data (*n* = 31) were acquired using a 3.0T Philips Achieva MRI scanner. The mean (±SD) age of the individuals was 34.5 ± 9.4 years (age range: 22–61 years).
3. Johns Hopkins University internal 3T axial data: These data (*n* = 50) were also acquired using a 3.0T Philips Achieva MRI scanner. The mean (±SD) age of the individuals was 61.1 ± 12.4 years (age range: 30–83 years).
4. International Consortium for Brain Mapping 1.5T axial data (RRID:SCR_001948): These data (*n* = 26) were acquired using a 1.5T Siemens Sonata MRI scanner. The mean (±SD) age of the individuals was 42.1 ± 12.1 years (age range: 20–68 years).

A detailed analysis of the effects of protocol variability on our segmentation pipeline has been published elsewhere (20).

### Data Sources: Patient Data

We used a database of clinically acquired MRIs from 92 patients with memory problems who had visited the Bayview Medical Center, Memory and Alzheimer's Treatment Center (Baltimore, MD). Data were accessed and analyzed with a waiver of informed consent by the Johns Hopkins Institutional Review Board. MRI scans followed the ADNI protocol, with three-dimensional MPRAGE, scanned by a 3T scanner (Siemens Verio).

### Anatomical Knowledge Filter by Multi-Atlas Segmentation

**Figure 1:**
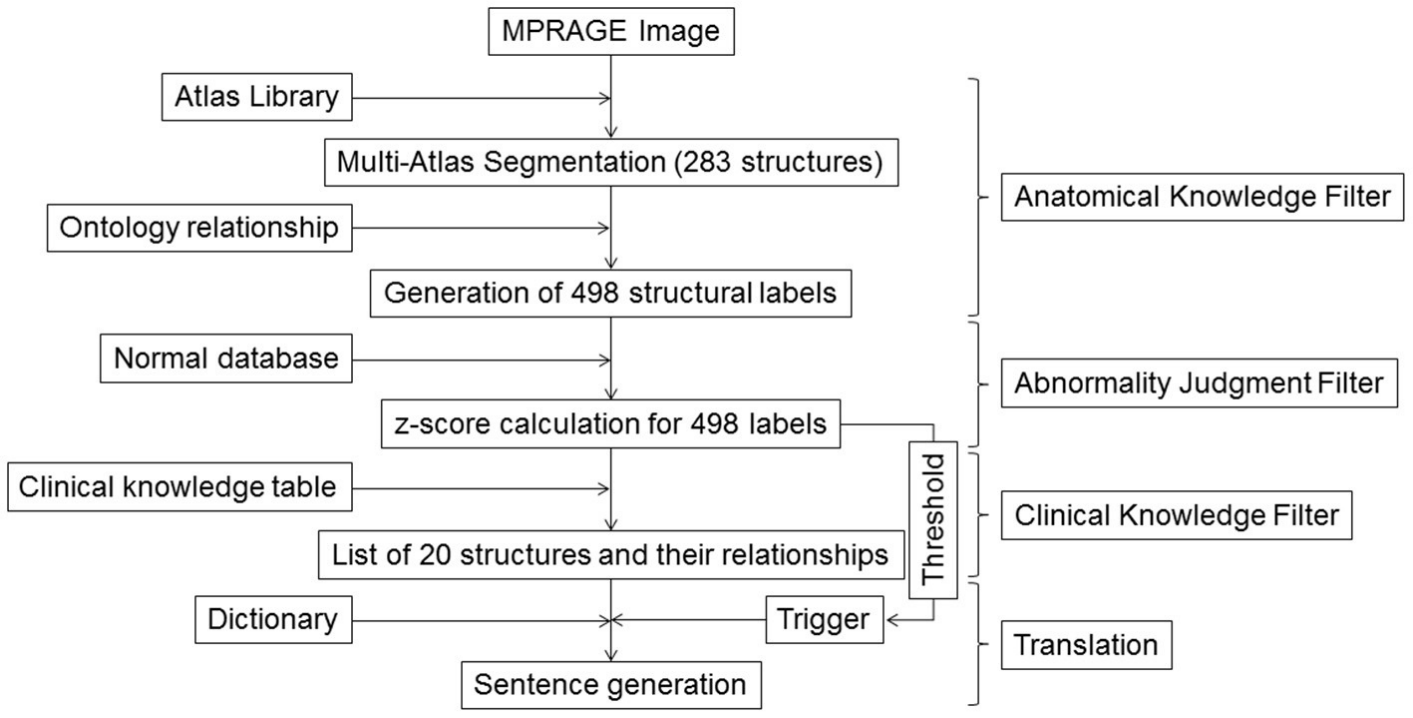
Schematic diagram for the image–sentence conversion. MPRAGE images were first segmented into 283 structural units using a multi-atlas segmentation tool, from which an additional 215 superstructures were defined. This process of reducing an image with >10^6^ voxels to 498 structures was called the *Anatomic Knowledge Filter*. In the second step, called the *Abnormality Judgment Filter*, volumes of structures were compared with values retrieved from databases of healthy individuals, with z-scores (criteria for abnormality judgment) calculated. In the third step, z-score information for the 498 structures was reduced to information on 20 selected structures and their relationships, using a *Clinical Knowledge Filter*. The final step was the translation of this information to human-readable sentences using a dictionary. MPRAGE, magnetization-prepared rapid gradient-echo.

The steps used to generate sentences are shown in Figure 1. In the last two decades, technologies for quantitative image analyses have significantly advanced (10, 21–25). One of the most commonly used quantitative analyses is voxel-based analysis (26, 27) that identifies statistically abnormal voxels in a fully automated manner. The voxel-by-voxel results are based on standardized anatomic coordinates with more than 7 million voxels. The voxel-based results, however, do not directly carry semantic labels and the sheer number of the voxels makes the anatomical and clinical interpretation highly challenging. In this study, we applied automated whole-brain segmentation as the first step. We can consider this step as a knowledge-driven data-reduction (thus called Anatomical Knowledge Filter) from a >10^6^ voxel domain to a much smaller number of a structure domain, because the manner of voxel aggregation to the structures is dictated by anatomical knowledge defined in reference atlases.

We used a modern multi-atlas approach for the segmentation involving more than 50 atlases (28) and covering an age range of 20–95 years. These atlases were warped to individual patient images, followed by an arbitration process to reach the final segmentation results. For the multi-atlas segmentation, we used the MRICloud (www.mricloud.org) pipeline, which is based on a previously described method (29–33). The current Johns Hopkins University multi-atlas library (version 9.b) identifies 283 anatomic structures.

Although conversion of the >10^6^ voxel dimension to the 283 structures would lead to substantial spatial data-reduction, existing RRs show that it is common to use coarser anatomic notions such as “tissue,” “parenchyma,” “global,” “hemisphere,” “lobes,” and “ventricles.” To generate the larger anatomic representations that are widely used in RRs, we adopted the flexible granularity control tool described in our previous publication (34). Briefly, multiple levels of superstructures were created based on multiple levels of ontology-based hierarchic relationships and applied to the 283 structures. In all, 498 structures were defined with all levels combined.

### Judgment of Abnormality

The second step was to define normal ranges for all 498 structures. For each structure, normal values were defined based on the 179 control scans. Age-corrected mean and SD values were calculated from which z-scores (= [measured volume–age-matched mean volume]/standard deviation) were estimated. The age-correction was performed for each segmented structure using linear regression. We have previously examined both raw volume values and volume ratios normalized by total brain volume (i.e., addition of all tissue segments, ventricles, and sulci), while the definition of the sulci includes only the deep part. We employed volume ratios because the variability of the normal values was significantly smaller than that of raw volume values, although the best way to calculate z-scores may remain a subject for debate.

### Clinical Knowledge Filter, Dictionary, and Sentence Triggering

Once the 498 structures were defined, it was possible to report the volumes and z-scores of all structures to the radiologists. However, this would certainly not be useful for routine clinical support. From the clinical point of view, not all structures are equal. One of the roles of the *Clinical Knowledge Filter* was to select structures with likely clinical importance from the 498 defined labels. Supplementary Table 1 lists the 20 selected structures used in this study.

#### Direct Triggering

For each of the 20 structures, the translator could generate a sentence based on the z-score. For example, if the z-score of the left hippocampus was smaller than a threshold:

➢ *If z-score(hippocampus_L)*<*-2.0, then trigger, “The left hippocampus has atrophy.”*

While we could have used only direct triggering for the 20 structures, we tested two more advanced translator classes to enhance anatomic interpretation. This is because radiologists may observe brain structures based on comparisons and combinations to extract clinically meaningful observations, rather than independent entities.

#### Relational Analysis

Three types of relationships among structures were examined, which triggered specific sentences:

1. Left vs. right: The terms “bilateral,” “left,” and “right” were used based on the relationship between the right and left counterparts. For example, at the ontology Level 1 definition, there were two anatomic labels: “hemisphere_L” and “hemisphere_R.” Using these labels, the following sentences could be triggered based on the z-scores:
  ➢ *If “hemisphere_L”*<*-2.0 AND “hemisphere_R”*<*-2.0, then “There is bilateral hemispheric atrophy”*
  ➢ *If “hemisphere_L”*<*-2.0 AND “hemisphere_R”*>*-2.0, then “There is left hemispheric atrophy”*
2. Hemispheric vs. lobar: When hemispheric atrophy was observed, the atrophy of the five constituent lobes was examined. If the hemispheric atrophy was localized in a small number of lobes, a nested sentence that included “prominent” was generated using nested Boolean operations:
  ➢ *If “hemisphere_L”*<*-2.0 AND “hemisphere_R”*>*-2.0, then “There is left hemispheric atrophy”*
  ° *If “frontal_L”*<*-2.0 AND “temporal_L”*>*-2.0 AND “parietal_L”*>*-0.2 AND “occipital_L”*>*-2.0 AND “limbic_L”*>*-2.0, then “The atrophy is prominent in the frontal lobe”*
3. Relationships among lobes: If there was no hemispheric atrophy, but atrophy was found in a small number of lobes, a sentence that included “specific” was generated:
  ➢ *If “hemisphere_L”*>*-2.0 AND “hemisphere_R”*>*-2.0 AND “frontal_L”*<*-2.0 AND “parietal_L”*>*-2.0 AND “temporal_L”*>*-2.0 AND “occipital_L”*>*-2.0 AND “limbic_L”*>*-2.0 AND “frontal_R”*>*-2.0 AND “parietal_R”*>*-2.0 AND “temporal_R”*>*-2.0 AND “occipital_R”*>*-2.0 AND “limbic_R”*>*-2.0, then “There is left frontal lobe specific atrophy”*

#### Combinations of Structures

Atrophy in certain lobar combinations was selected to trigger sentences if such combinations were known to have clinical significance. For example, frontotemporal dementia frequently involves atrophy in the left frontal and temporal lobes. Assessing this would require an examination of the relationship among the frontal, parietal, occipital, temporal, and limbic lobes, such as:

➢ *If “hemisphere L”*>*-2.0 AND “hemisphere R”*>*-2.0 AND “frontal_L”*<*-2.0 AND “temporal_L”*<*-2.0 AND “parietal_L”*>*-0.2 AND “occipital_L”*>*-2.0 AND “limbic_L”*>*-2.0 AND “frontal_R”*>*-2.0 AND “temporal_R”*>*-2.0 AND “parietal_R”*>*-0.2 AND “occipital_R”*>*-2.0 AND “limbic_R”*>*-2.0, then “There is left front-temporal lobe specific atrophy”*

Note that this sentence was not triggered when there was hemispheric-level atrophy due to the inclusion of “*If ‘hemisphere_L'*>*-2.0 AND ‘hemisphere_R’*>*-2.0*.” For example, if the right hemisphere had significant atrophy, the sentence with “*left front-temporal lobe specific*” would not be triggered because the atrophy would no longer be specific to the left frontal and temporal lobes.

With the five major lobes defined in this study, there were 30 different combinations of lobar atrophy patterns in addition to four patterns of hemispheric atrophy. The relational and combination triggering classes were used to avoid a large number of direct triggerings and efficiently draw the radiologists' attention to clinically important features. Our initial attempt to create the Boolean operations is described in Supplementary Table 2. The *Clinical Knowledge Filter* is a work in progress; hence, evaluation of its efficacy was an important purpose of this paper.

### Evaluation by Subjective Assessment

**Figure 2:**
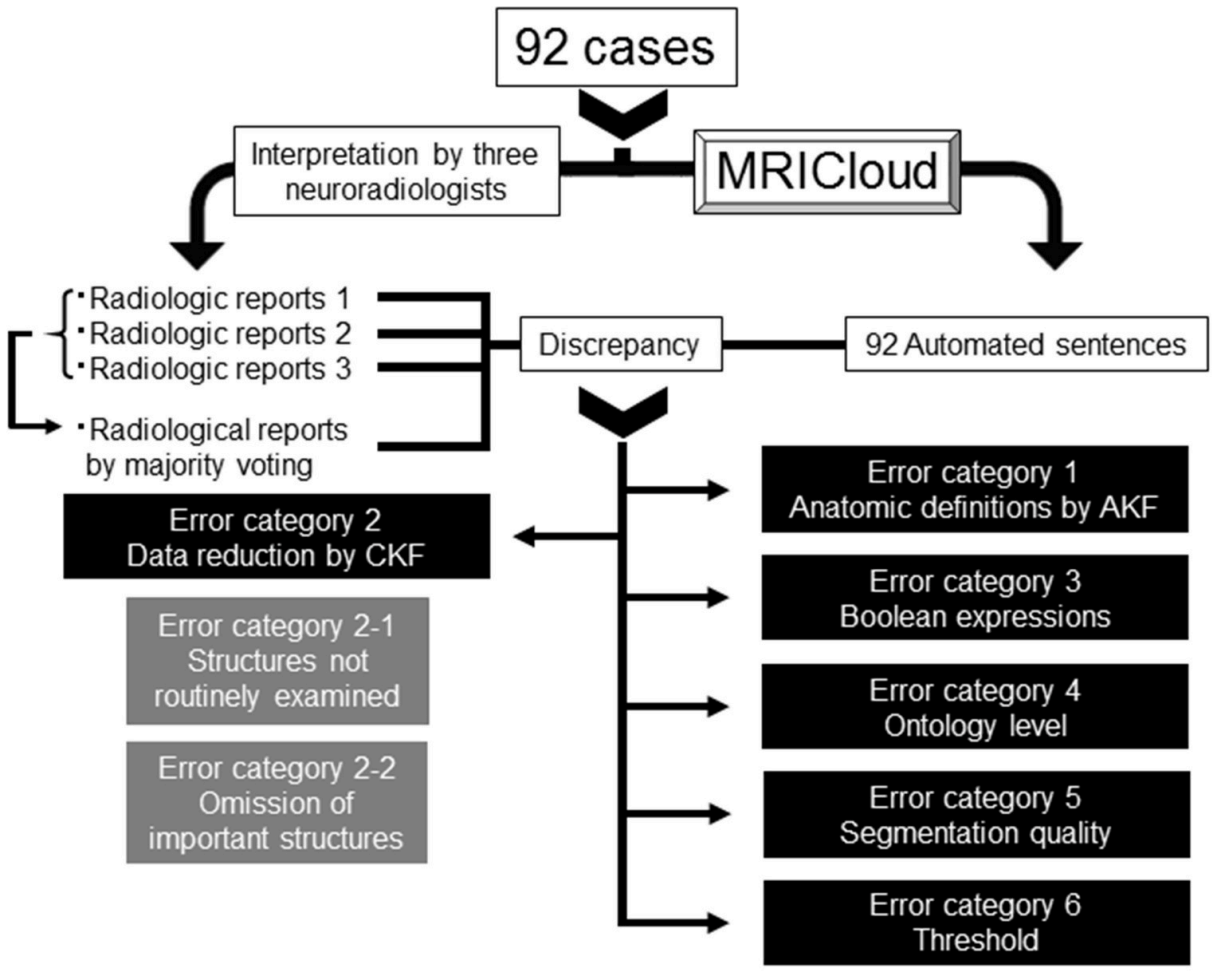
Decision-making tree of the reasons for discrepancies between the radiologic reports and automated sentences. AKF, Anatomic Knowledge Filter; CKF, Clinical Knowledge Filter.

This was a retrospective study based on existing data. The 92 images from the patients with memory problems were assessed by three neuroradiologists with more than 6 years of experience and without prior knowledge of the automated analyses. They were informed of the patients' age and the fact that dementia was suspected by attending physicians. They are instructed to read the images and generate radiological reports in ways the routinely do in their daily clinical practices without standard templates. The outcome was a free-text RR. For each sentence, agreement between the automated sentence (AS) and the RR, as well as between the RRs, were tested by KA and categorized as true-positive (TP), false-negative (FN), false-positive (FP), or true-negative (TN) case. In the case of disagreement (FN + FP), its reasons were carefully examined. We first examined if there were differences in agreement depending on the structure type: brain tissues and non-brain tissue (ventricles and sulci) structures. Then, for brain tissue structures, we systematically studied the reasons for discrepancy and categorized the errors into six classes using the decision-making tree described in Figure 2. The raw data supporting the conclusions of this manuscript will be made available by the authors, without undue reservation, to any qualified researcher.

#### Error Category 1: Anatomic Definitions by the Anatomical Knowledge Filter

On the basis of the 283 defined structures, further 215 additional superstructures were defined (total 498 structures). While this provided an extensive list of anatomic names, it is still possible that radiologists use anatomic names that were not defined in our atlas. In theory, this could happen in one of three ways: (1) anatomic entities that were smaller than the smallest units defined in the atlas were used; (2) the anatomic borders of the structural notions used by the radiologists did not follow the anatomic definitions used in our atlas; and (3) none of the superstructures represented the anatomic terms used by the radiologists. In these instances, it was not possible to evaluate agreement. We assigned these cases to error category 1.

#### Error Category 2: Errors in the Information Reduction by the Clinical Knowledge Filter

Of the 498 structures defined, only 20 were chosen for this study through the *Clinical Knowledge Filter*. This knowledge filter was developed because radiologists implicitly give heavier weightage to certain structures than others. For example, the target population of this study was patients with memory problems, and radiologists therefore paid special attention to the hippocampus. This weighting resulted in certain structures being frequently described, while others were rarely mentioned. It is not clear that the choice of 20 structures was appropriate, and the following patterns of errors might have occurred.

*Error category 2-1: inclusion of structures that were not routinely examined:* It is possible that the ASs would include sentences about structures that were rarely seen in the RRs. This would involve the triggering of sentences that a radiologist might judge unnecessary. This can be considered an FP result. With the 498 defined structures (and their relationships), we could generate large numbers of “scientifically correct” sentences about anatomic states. However, such an approach would be unacceptable in practice. In this sense, knowledge-driven reduction of the list was necessary but at the cost of generating this error category.
*Error category 2-2: omission of important structures from the knowledge-based dictionary:* As opposed to error category 2-1, the knowledge-based reduction may remove clinically important labels, which lead to FN cases and are categorized in this class.

#### Error Category 3: Errors in the Boolean Expressions in the Translator

The relationships defined in the *Clinical Knowledge Filter* often became complicated, and we experienced unexpected triggering or omission of important observations. These cases were documented and categorized in this class.

#### Error Category 4: Ontology-Level Errors

A significant number of discrepancies were caused by the extent of areas of abnormalities referred to by the AS and the radiologists. For example, if the AS described “The left hemisphere has atrophy” and a radiologist described “The left front-temporal lobe has atrophy,” then such a case was included in this error category. Obviously, there is ambiguity in the definition of this error; in the above example, the radiologist may not mean “atrophy is restricted ONLY in the left frontal and temporal lobe.” However, the computer-generated sentences require rigorous definition of anatomical specificity for triggering of each sentence, and atrophy in the “left hemisphere” and “left front-temporal lobe” have different triggering criteria. Although this may not be considered as clear errors of the AS, we categorized them in this error class.

#### Error Category 5: Poor Segmentation Quality

If a discrepancy was not categorized in error categories 1–4, we carefully examined the segmentation results to examine whether the error was caused by segmentation errors. If one of the radiologists judged that the discrepancy was caused by a segmentation error, the case was classified as error category 5.

#### Error Category 6: Inadequate Threshold for Triggering (Judgment Filter)

If a discrepancy could not be classified into error categories 1–5, the radiologists were asked if they felt that the threshold to trigger the AS with a z-score smaller or larger than 2.0 was too stringent or too relaxed with resulting errors placed in this category.

## Results

### Overall Agreement Based on Structure Types

Table 1 summarizes the agreement between the ASs and RRs for brain tissue and non-brain tissue (ventricles and sulci) structures. For the 92 patients analyzed, we categorized the findings into TP, FP, FN, or TNs simply based on whether any comments were made in the RRs about the brain tissue or non-brain tissue structures, regardless of location. For example, the ASs and radiologist did not mention any brain tissue abnormalities for 20 patients, while they both described abnormalities in 45 patients. The number of the FP and FN cases was similar, indicating that the sentence-trigger threshold used in this study (z-score <-2.0) was reasonable. The accuracy of the AS (mean 0.70), sensitivity (mean 0.80), precision (mean 0.71), and specificity (mean 0.60) were mediocre. These numbers do not have much clinical meaning, however, because they do not represent exact agreement with respect to the locations of abnormal brain tissue structures.

**Table 1:** The number of the positive and/or negative findings in the brain tissue and the ventricle among AS and 3 RRs.

**Region**	**AS**	**RR**			
			**RR1 (*n*)**	**RR2 (*n*)**	**RR3 (*n*)**
Brain tissue	Positive	Positive	45	32	45
	Positive	Negative	12	25	12
	Negative	Positive	15	4	15
	Negative	Negative	20	31	20
		Accuracy	70.7%	68.5%	70.7%
		Sensitivity	75.0%	88.9%	75.0%
		Precision	78.9%	56.1%	78.9%
		Specificity	62.5%	55.4%	62.5%
Ventricle	Positive	Positive	5	5	3
	Positive	Negative	30	30	32
	Negative	Positive	0	0	0
	Negative	Negative	57	57	57
		Accuracy	67.4%	67.4%	65.2%
		Sensitivity	100.0%	100.0%	100.0%
		Precision	14.3%	14.3%	8.6%
		Specificity	65.5%	65.5%	64.0%

*AS, Automated sentence; RR, Radiologic report*.

Comments about ventricles and sulci were separated from those about brain tissue structures for several reasons. First, all comments about ventricles were about enlargement (no cases of abnormally small ventricles were reported), while all comments about brain tissues were about size reductions. Second, RRs from all three radiologists reported ventricular abnormalities when they were severe. In mild-to-moderate cases, RRs tried to describe the brain tissue structures that accounted for the ventricle enlargement. The mean z-score of the RR-positive cases was 5.45 ± 1.5, considerably higher than the threshold used in the AS. As a result, agreement among the reports about ventricles had a sensitivity of 1.0 (i.e., when the RR described abnormal ventricles or sulci, the AS always accurately detected these as abnormal), but precision (RR positive/AS positive cases), and specificity (AS negative/RR negative) was low (mean of 0.12 and 0.65, respectively). The remaining Results focus on reports of brain tissue structures.

### Detailed Analyses of Agreements and Disagreements for Brain Tissue Structures

Structure-by-structure agreements and disagreements are presented in Table 2. The current AS generator includes 16 specific brain tissue names (see Supplementary Table 1). Seven additional anatomic structures were mentioned by radiologists. With 92 cases with memory problems, there were a total of 2,116 potential entries (92 cases × 23 structures) for which structure-to-structure agreement was to be examined (TP, FP, FN, and TN cases). The results show high agreement (90.9–92.1%) between the ASs and the three radiologists (radiologists 1, 2, and 3), while the mean inter-rater agreement was 92.3%. The accuracy increased to 93.1% between the ASs and the majority voting (judgment based on agreement by more than 2 raters), while the mean level of agreement between the radiologists and the majority voting was 96.1%. Although these numbers seem promising, they were driven by the large number of TN cases, artificially increased by adding reports about many structures that were not affected in this patient population. As seen from the low mean sensitivity (24.5%), precision (24.9%), and kappa (0.20) values, the performance of the AS should be considered poor. Because sensitivity, precision, and kappa values are less affected by the number of TN cases, we focus on these three measures in the following sections.

**Table 2:** The number of positive and/or negative findings of all anatomic structures that were mentioned in AS or RRs among AS, 3 RR, and RR by majority voting.

	**AS-3 RR**				**AS-RRm**		**RR-RR**				**3 RR-RRm**			
	**AS-RR1**	**AS-RR2**	**AS-RR3**	***Mean***			**RR1-RR2**	**RR1-RR3**	**RR2-RR3**	***Mean***	**RR1-RRm**	**RR2-RRm**	**RR3-RRm**	***Mean***
TP (n)	23	31	34	*29.3*	28	P-P (n)	39	36	42	*39.0*	59	64	61	*61.3*
FP (n)	94	86	85	*88.3*	89	P-N (n)	82	85	79	*82.0*	64	58	58	*60.0*
FN (n)	98	90	83	*90.3*	56	N-P (n)	82	83	76	*80.3*	25	20	23	*22.7*
TN (n)	1901	1909	1914	*1908.0*	1943	N-N (n)	1913	1912	1919	*1914.7*	1968	1974	1974	*1972.0*
Accuracy	90.9%	91.7%	92.1%	*91.6%*	93.1%	PP/Total	92.2%	92.1%	92.7%	*92.3%*	95.8%	96.3%	96.2%	*96.1%*
Sensitivity	19.0%	25.6%	29.1%	*24.5%*	33.3%	PP/(PP+NP)	32.2%	30.3%	35.6%	32.7%	70.2%	76.2%	72.6%	73.0%
Precision	19.7%	26.5%	28.6%	24.9%	23.9%	PP/(PP+PN)	32.2%	29.8%	34.7%	32.2%	48.0%	52.5%	51.3%	50.5%
Specificity	95.3%	95.7%	95.7%	*95.6%*	95.6%	NN/(NN+PN)	95.9%	95.7%	96.0%	95.9%	96.9%	97.1%	97.1%	97.0%
kappa value	0.15	0.22	0.25	0.20	0.24	kappa value	0.28	0.26	0.31	0.28	0.55	0.60	0.58	0.58

*AS, Automated sentence; N, Negative; P, Positive; RR, Radiologic report; RRm, RR by majority voting*.

The inter-rater sensitivity and precision rates were 30.3–35.6% and 29.8–34.7%, respectively. The kappa values were 0.26–0.31. These values are higher than AS–RR agreement, but still low. The agreement rates depend heavily on the strictness of the agreement criteria, especially about the location. In this study, we employed rather strict criteria (e.g., left hemispheric atrophy and left frontal-temporal atrophy were not in agreement). The reported levels of agreement can be compared among different approaches within this study but care must be taken in comparison with those from different studies. An improvement with majority voting was obvious, with sensitivity and precision increasing to 73.0 and 50.5%, respectively. Considering that the AS vs. majority voting agreement was only 33.3% (sensitivity) and 23.9% (precision), overall, the performance of the ASs was inferior to that of the RRs.

### Error Categorization

Table 3 shows the reasons for disagreements using the six error categories.

**Table 3:** Number and percentage of each error category and subcategory.

	**Error (n)**	**Category 1**		**Category 2**						**Category 3**	**Category 4**	**Category 5**	**Category 6**	
		**Global**	**Medial temporal**	**2-1**				**2-2**					**False positive**	**False negative**
				**Limbic**	**Parietal**	**Occipital**	**Caudate**	**Cerebellum**	**Amygdala**					
RR1	192	34 (17.7%)		10 (5.2%)				16 (8.3%)		0 (0.0%)	29 (15.1%)	23 (12.0%)	80 (41.7%)	
		15 (7.8%)	19 (9.9%)	3 (1.6%)	3 (1.6%)	1 (0.5%)	3 (1.6%)	6 (3.1%)	10 (5.2%)				56 (29.2%)	24 (12.5%)
RR2	176	7 (4.0%)		10 (5.7%)				2 (1.1%)		11 (6.3%)	14 (8.0%)	14 (8.0%)	118 (67.0%)	
		3 (1.7%)	4 (2.3%)	3 (1.7%)	3 (1.7%)	1 (0.6%)	3 (1.7%)	2 (1.1%)	0 (0.0%)				62 (35.2%)	56 (31.8%)
RR3	168	0 (0.0%)		10 (6.0%)				13 (7.7%)		5 (3.0%)	3 (1.8%)	16 (9.5%)	121 (72.0%)	
		0 (0.0%)	0 (0.0%)	3 (1.8%)	3 (1.8%)	1 (0.6%)	3 (1.8%)	0	13 (7.7%)				72 (42.9%)	49 (29.2%)
RRm	145	4 (2.8%)		10 (6.9%)				5 (3.4%)		2 (1.4%)	10 (6.9%)	18(12.4%)	96 (66.2%)	
		2 (1.4 %)	2 (1.4%)	3 (2.1%)	3 (2.1%)	1 (0.7%)	3 (2.1%)	2 (1.4%)	3 (2.1%)				67 (46.2%)	29 (20.0%)

*RR, Radiologic report, RRm, RR by majority voting*.

#### Category 1 Errors

Two anatomic terms were categorized in this error type: “medial temporal lobe” and “global.” The term “medial temporal lobe” was used 19 times by radiologist 1 (9.9% of the total errors) and four times by radiologist 2 (2.3%). This term is widely used in radiology and neurology, and is usually meant to include the hippocampus, entorhinal cortex, parahippocampal gyrus, and sometimes associated regions (perirhinal and fusiform gyrus), as well as the amygdala. The term, “medial temporal” also implies that these structures are part of the temporal lobe. On the other hand, in ontological definitions, many of these structures are integrated into the limbic system as a superstructure together with the cingulate gyri. Our anatomic definitions followed the latter definition, and “medial temporal lobe” was not defined.

The term “global” includes the hemispheres, cerebellum, and brainstem, encompassing a larger anatomic entity than the largest superstructure we used (i.e., the hemispheres). This term was used 15 times by radiologist 1 (7.8% of the total errors) and three times by radiologist 2 (1.7%).

#### Category 2 Errors

Errors in category 2-1 were minor, responsible for only 5.2–6.0% of errors. Among them, four structures selected in the *Clinical Knowledge Filter*—the limbic system, parietal lobe, occipital lobe, and caudate nucleus—were not evaluated by any of the radiologists. As these terms were not mentioned by the radiologists, all positive cases generated by the ASs were classified as FPs.

Errors in category 2-2 included two anatomic names—“cerebellum” and “amygdala”—mentioned in the RRs, but eliminated in the *Clinical Knowledge Filter* and thus not included in the ASs. These cases were classified as FN. Error category 2-2, however, constituted only 3.4% of the majority voting, suggesting inconsistencies among radiologists in the use of these anatomic names in reports.

#### Category 3 Errors

Category 3 errors were also minor, comprising 0–6.3% of the total disagreement with radiologists 1–3 and only 1.4% of the majority voting. The causes of these errors were unique to the nature of the relationship analyses. In this category, all structures that the radiologists reported as abnormal were consistent with the quantitative results (z-score <-2.0), but the system did not generate ASs that agreed with the regional specificity described by radiologists. For example, in one of the cases, RRs described atrophy in the left frontal lobe. This observation was correctly supported by the quantitative results (left frontal lobe <-2.24). However, this observation was not triggered by the AS generator because the Boolean operation would not generate a report specific to left frontal lobe atrophy unless atrophy was confined to the left frontal lobe. The analysis of the relationships among and combinations of two hemispheres and five lobes can be complicated and, although minor, unexpected difficulties in the Boolean operation errors were encountered.

#### Category 4 Errors

The errors in this category constituted 1.8–15.1% of the errors for the three radiologists and 6.9% for majority voting. The most frequent discrepancy was caused by ASs reporting hemispheric atrophy with RRs reporting atrophy in the global brain or frontal and temporal lobes. Other frequent discrepancies included “hemispheric atrophy” vs. “global brain atrophy” and “temporal lobe atrophy” vs. “medial temporal atrophy” for ASs vs. RRs.

#### Category 5 Errors

**Figure 3:**
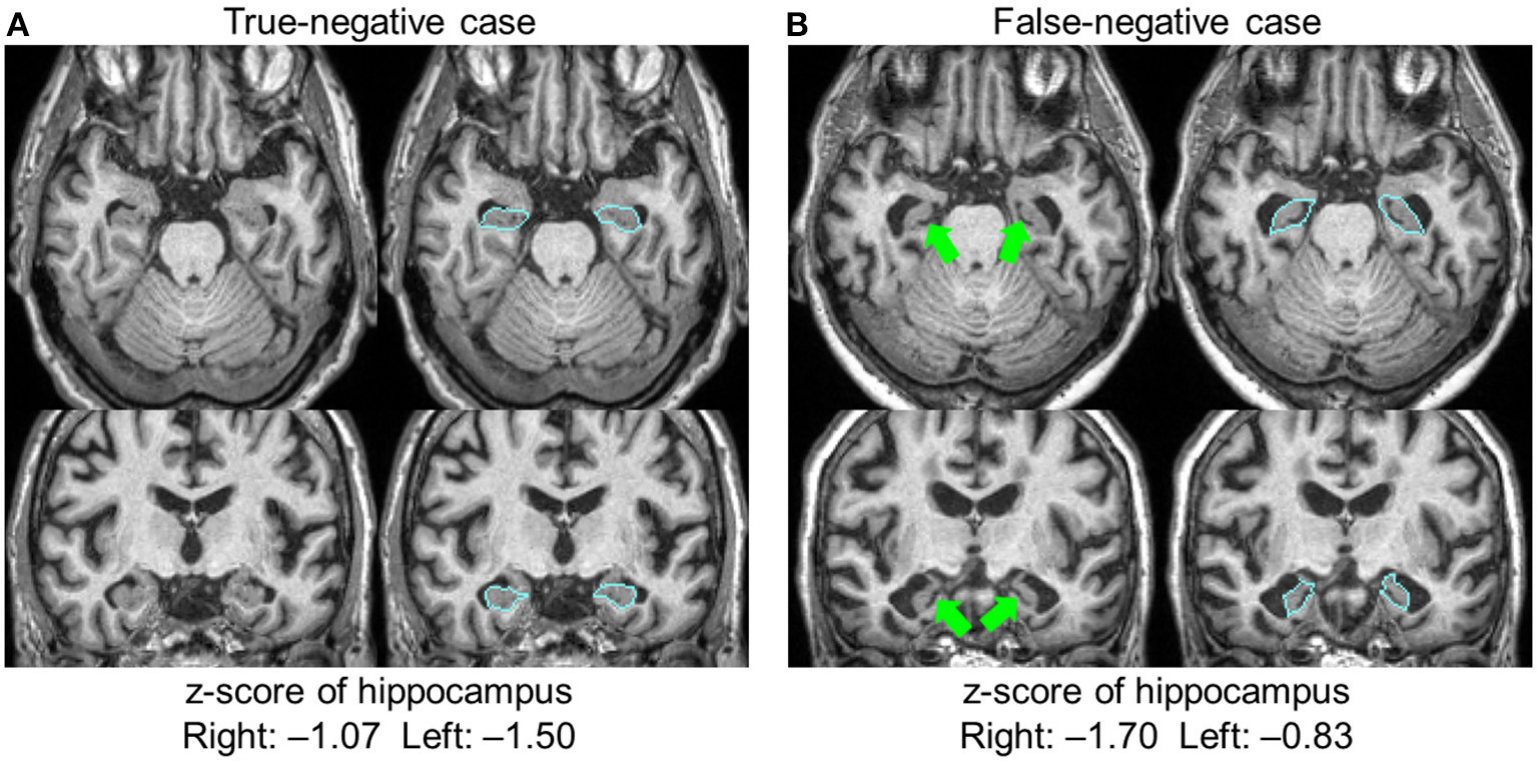
Two examples of hippocampal segmentation. Both cases had a similar degree of atrophy based on the quantification results, with z-scores higher than −2.0; both were judged as normal and automated sentences were not triggered. The radiologists agreed that Case **(A)** was normal (true-negative), but Case **(B)** was judged to be atrophic (false-negative). In this case, the opening of the uncal sulci (arrows) was a factor in the radiologists' judgment. Blue lines show the boundary of the hippocampus.

The hippocampus was the only structure in which segmentation errors were judged to be the cause of the AS–RR discrepancy, with errors regarding 24 hippocampi in 15 patients. These cases were all labeled as FN (ASs were negative but RRs noted atrophy). Among them, there was one case with an apparent leak to the choroid plexus, which caused the FN (the leak to the choroid plexus caused a z-score>-2). In other cases, the radiologists reported leakages, but the extent of the leaks was small and did not affect sentence-triggering. There were no cases wherein radiologists thought the hippocampus was erroneously defined as smaller than its actual size. The remaining 23 hippocampi had consistent anatomic features that caused the FN results. Figure 3 shows two examples of hippocampus segmentation, along with the quantification results. Case A is an example of a TN case, while Case B is an example of an FN case. What was common to all 23 FN cases was the partial opening of the uncal sulcus. With the segmentation algorithm used in this study, this sulcus was included as a part of the hippocampus unless it was completely open with respect to the imaging resolution (>1 mm). The radiologists, however, used appreciable opening of the uncal sulcus as an indicator of hippocampal atrophy.

#### Category 6 Errors

**Figure 4:**
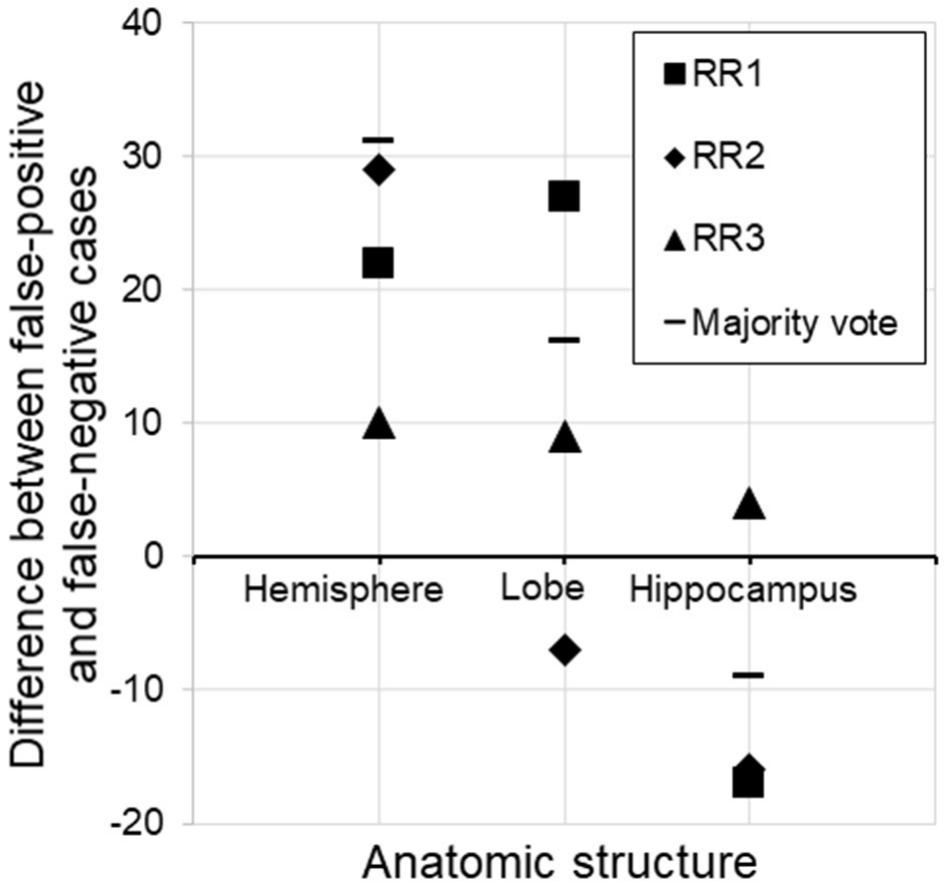
Subtraction of false-negative cases from false-positive cases for three different structures. For the hemisphere, there were more false-positive than false-negative cases, suggesting that the threshold (z-score <-2.0) was too low (too close to zero and sentences were triggered too often). On the other hand, the same threshold was too relaxed for the hippocampus, assuming the radiologists' judgment to be the gold standard. RR, radiologic report.

By far, this was the largest error category, wherein errors were caused by the choice of threshold for AS triggering (z-score>2.0 or <-2.0). These errors constituted 41.7–72.0% of the total errors. The overall FP/FN ratios owing to the threshold were 56/24, 62/56, and 72/49 for RR 1–3, respectively, and 67/29 for majority voting. Overall, this implies that the choice of an absolute z-score of 2.0 was too low (too close to zero and sentences were triggered too often). However, closer inspection revealed a more complex view of this issue (Figure 4). First, it appears that the appropriate threshold might vary depending on the structure being examined, with the radiologists tending to apply less stringent thresholds to larger structures. Second, the threshold used was seen to vary among the radiologists.

## Discussion

### Using RRs as the Gold Standard

In this paper, we report the results of our first attempt to automatically convert raw T1-weighted MRI images into a human-readable summary of clinically important features. We treated the RRs as the gold standard to judge the performance of AS. This is because of the fact that the subjective RRs are what accepted in the routine clinical practices and thus, if there was discrepancy between a radiologist and an automated report, we declared that it was an error by the automated report. The validity of this approach (using RRs as the gold standard) is controversial. Therefore, we made this study as descriptive as possible without trying to prove the superiority of one to the other. The alternative approach would be data-driven, in which the performance is judged from data; in this study, the diagnostic accuracy could be one of the criteria. However, as for many clinical observations with various medical conditions, MRI of patients with memory problems is only a piece of medical information that could estimate the pathology and prognosis at a given time point, and could change over the course of disease progression. Therefore, it would be difficult to interpret if AS is superior to RRs or vice versa. Although the knowledge-driven design also has weaknesses, this could be a step toward our deeper understanding of how computers can assist clinical practices.

### Accuracy of Segmentation

Assuming that we can use RRs as the gold standard, probably one of the most interesting questions is “How accurate are the computer-generated sentences?”; the accuracy of the brain segmentation is without doubt the most crucial factor. However, for several reasons, segmentation accuracy is not the center of interest in this study. First, with the rapid development of brain segmentation technology in the past decade, particularly with respect to multi-atlas approaches, the segmentation accuracy of recent tools is adequately high to analyze clinical data with confidence. In this study, all three radiologists felt the overall segmentation accuracy of the ASs was high, except for those listed under error category 5. Second, the segmentation tool can be readily replaced when better tools become available.

Category 5 errors were dominated by the hippocampus. This is probably because the hippocampus is the smallest structure among those evaluated in this paper, and even a small amount of leakage to adjacent structures may be significant. Except for the hippocampus and the caudate, all other structures included in the *Clinical Knowledge Filter* were superstructures of much larger sizes, and there were no cases that the radiologists labeled as segmentation errors.

The concept of accuracy requires a gold standard, which, in the field of neuroanatomy, is defined by human perception. This poses a fundamental difficulty for accuracy and validation studies of neuroanatomy. In reality, a certain level of disagreement between automated segmentation and human validation is expected. However, we found a very important discrepancy with respect to the interpretation of the uncal sulcus opening (Figure 3). Although it is difficult to declare that our segmentation results for Case B represented an obvious error, the fact that this sulcus became visible is an important indicator for radiologists in judging the degree of atrophy.

### Difficulties in Data Reduction Based on Anatomic Knowledge

During the conversion of raw image matrices to semantic representations with clinical importance, data-reduction is necessary. In the first reduction of an image with >10^6^ voxels to the total 498 structures, we encountered two structures that were undefined: “medial temporal lobe” and “global” that were used by two radiologists. This led to category 1 errors, which might be solved by defining a superstructure that represents these two structures. This suggests that the list of anatomic structures defined in our atlas was comprehensive enough to cover most clinical terminology referring to specific anatomic regions. On the other hand, reports about 498 anatomic structures would be impractical for daily clinical practice and may mask clinically important findings. Further steps of information reduction are therefore required.

#### Weighting by Clinical Knowledge

Instead of providing descriptions about 498 defined structures, we could select those known to be clinically important. This weighting could be disease-dependent. In this study, a *Clinical Knowledge Filter* was designed for patients with memory problems by using three such filters.

#### Simple Selection Filter

Out of 498 defined structures, we selected 20 that we believed to be important in this population. Inclusion of structures not mentioned by radiologists and exclusion of structures that were mentioned were categorized as type 2-1 or 2-2 errors, respectively.

This initial clinical knowledge filter was developed by reviewing past radiological reports and through discussions among four participating radiologists about “which types of anatomical structures are routinely evaluated for elderly patients with memory problems.” Because this is about the human knowledge, it is difficult to establish the complete prior knowledge. One of the purposes of this study is to evaluate the soundness of this initial knowledge filter. Our study indeed showed that certain failures were due to the incompleteness of this initial knowledge (type 2 errors). We expect this knowledge filter could be improved through the learning process based on the type 2 errors. On the other hand, this type of knowledge-based approach could have limitations when we try to incorporate more elaborated knowledge for, for example, certain disease subtypes (e.g., knowledge filter specifically designed for fronto-temporal dementia group). For this end, data-driven analysis of existing clinical reports using natural language processing and machine learning may be needed in the future.

#### Filter Through Relational and Combination Analyses

With 20 selected structures, it would be simple to generate up to 20 sentences or a single sentence that lists all affected structures. This approach, however, might lead to an awkward sentence if, for example, all five left lobes and the left hemisphere had atrophy as well as other atrophic structures in the right side. In this example, our translator would streamline the sentence to “There is left hemispheric atrophy” or “There is bilateral hemispheric atrophy.” These evaluations were, however, found to be complicated; evaluation of spatial specificity and relationships among structures is essential for image reading, but even with the 20 selected structures, it was not a straightforward task. Difficulties in identifying regional specificity for specific brain functions or pathology have been described in the past (35, 36). Namely, if a region reaches a significant level based on a structure-by-structure analysis, how can we test if such a result is region-specific? For example, in our patient population, there were 29 cases with atrophy in the left temporal lobe with z-score <-2.0. Among them, 14 had the entire left hemisphere atrophy with z-score <-2.0 and 8 of them had atrophy in other lobes. In these cases, it is not straightforward to establish a statistical method to test the regional specificity so that we can declare “the patient has temporal lobe atrophy.”

In our study, descriptions of certain regional atrophy could be suppressed when there is atrophy in a larger structure within the same structural hierarchy. This led to more stringent criteria to trigger a sentence for regional atrophy. This approach, however, does not contain any weighting based on clinical significance. For example, the fronto-temporal atrophy pattern is known to be an important clue to infer the type of dementia. One can argue that atrophy of this pattern should be reported even if the entire hemisphere has a similar degree of atrophy. Further development of sophisticated engines for anatomical pattern analyses is needed for future studies.

### Abnormality Thresholds

**Figure 5:**
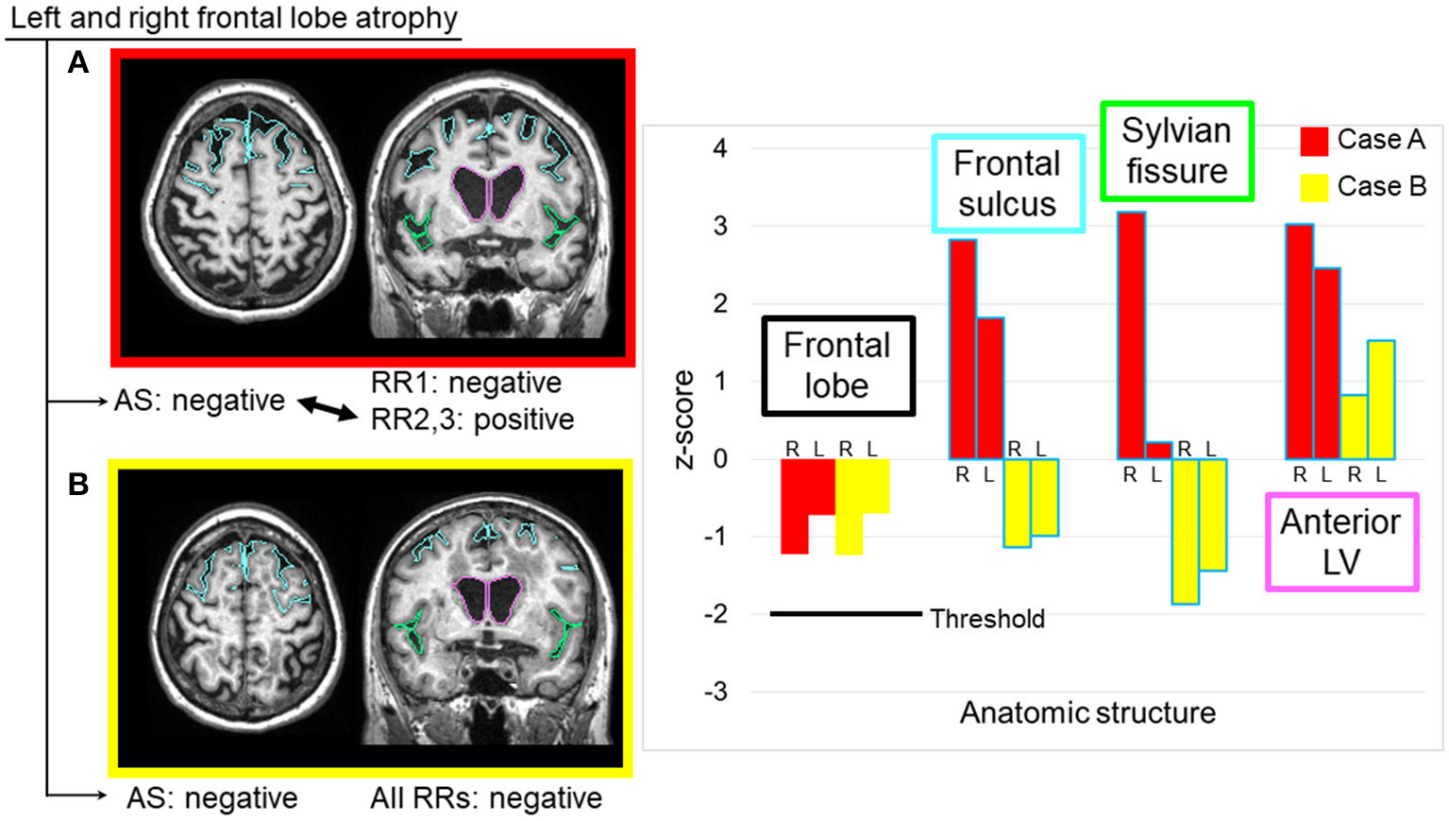
Two sample cases with similar z-scores for the frontal lobe volumes, but with different interpretations by radiologists. Two radiologists reported bilateral frontal lobe atrophy in Case **(A)**, where the image showed noticeable expansions of the bilateral frontal sulci and unilateral Sylvian fissure, as well as a highly dilated anterior lateral ventricle. All radiologists considered the frontal lobe to be normal in Case **(B)**, where the image showed relatively smaller frontal sulci and Sylvian fissures, and moderately dilated anterior lateral ventricles. AS, automated sentence; L, left; LV, lateral ventricle; R, right; RR, radiologic report.

In this study, the largest cause of errors was the threshold used to trigger sentences. We used a z-score of 2.0 as the AS threshold. In many cases, the radiologists felt that the threshold was too stringent, especially for the hemispheric and lobar-level structures, while being too relaxed for the hippocampus. Although we identified this structure-dependent tendency for threshold variability, there was also variability among the radiologists. Therefore, this issue cannot be resolved simply by changing the universal threshold level. It is also unclear whether different threshold values should be applied to particular structures. One interesting explanation for this variability is that, while quantitative analysis relies on the absolute volume of each structure, radiologists' judgments might include the anatomy of the surrounding structures. When the frontal lobe is examined, for example, the opening of the sulci and the enlargement of the lateral ventricles might provide important additional information. Figure 5 shows two cases with similar atrophy of the frontal lobes, indicating insignificant atrophy; however, in one of these cases, two radiologists reported bilateral frontal lobe atrophy. From the images in Figure 5, it is clear that these cases have very different atrophy patterns: one is accompanied by the opening of the frontal lobe sulci (z-score: right = 2.83, left = 1.82), while the other shows relatively tight sulci. The clinical significance of these two types of anatomic features is unclear, but routine clinical examination usually does not attempt to stratify these cases, while the state of the sulci might affect the subjective evaluation of the brain tissue atrophy.

Likewise, when examining the hippocampus, the uncal sulci and the inferior horn of the lateral ventricles provide important information (Figure 3). Another potentially significant contributor to this issue is age calibration. The same volumes of the hippocampus could indicate severe atrophy in a 60-year-old patient but be normal for an 80-year-old patient. In the quantitative analysis, age-dependency was corrected using the control database. The way in which radiologists subjectively corrected for age effects could introduce another source of variability.

A fundamental question that remains is the existence of threshold itself. Regardless of the adopted threshold, it is likely that radiologists cannot distinguish the degree of atrophy right above and below the threshold. This could be even more complicated when multiple structures are compared for spatial specificity such as left and right-dominant atrophy. This, however, also applies to radiological reports, in which radiologists apply implicit thresholds to trigger their descriptions. In this regard, this study provided interesting insight into radiologists' thought process by retrospectively investigating what were the threshold levels when radiologists declared atrophy. Although this is still a very descriptive phase of the research, we hope this would provide useful information to understand how human-machine interface should be created for medical judgement in the future, including a possibility to remove this type of threshold-based decision-making all together.

### Future Directions

In the future, it is possible that quantitative evaluation of morphological changes will be adopted in clinical practices. However, currently, it is not clear if radiologists would adopt such quantitative reporting by simply presenting quantitative volumes and statistical numbers, even if they are accurate. In this study, we shifted the human-machine interface one step closer to human by generating the sentences based on such quantitative measures.

To develop a tool that can automatically generate semantic reports, it is necessary to understand the thought processes of radiologists. This study revealed several interesting challenges to such efforts. A significant proportion was related to the *Clinical Knowledge Filter*, which converted volume data from 498 structures into sentences. Whether it is worth generating such reports depends on the benefit they provide. For example, if the generated sentences are provided when the images are read, radiologists could choose to agree with that information. However, whether this actually enhances the efficiency of clinical practice would need to be confirmed. Another important question is whether the generated sentences would improve the quality of reports. This depends on the experience of the radiologist and thus does not have a straightforward response. In this study, human-generated sentences were the gold standard, meaning that the performance of the AS generator could not be better than that of the radiologists. However, this study indicated inconsistency among radiologists. Quantitative tools have the potential to introduce a higher level of consistency across radiologists and institutions. This, however, is true only if the quantitative tools are robust against age effects, degree of pathology, and variability in imaging protocols, including hardware (20).

The above discussions bring to light another interesting question about the possibility of using image representation from the quantitative analyses, rather than sentence generation. In other words, if radiologists are presented with an image-based presentation of color-coded z-score data, then sentence generation might not be necessary. In this case, the role of automated analysis would be to present visual cues, to enhance the efficiency and consistency of image readings. This approach is far simpler than sentence-generation, and would negate the need for the *Clinical Knowledge Filter*. However, the burden of interpreting the quantitative images would remain with the radiologists. In past discussions with radiologists, we have often heard that the clinical interpretation of anatomic features is more demanding and important than the simple detection of atrophy. Therefore, sentence generation through the application of the *Clinical Knowledge Filter* ought to remain an important goal.

### Data-Driven Analysis Through Big Data Analytics

We have discussed the potential value of AS generation in improving radiologic workflow. Another interesting challenge would be to use these tools to perform large-scale analysis of medical records. This is highly relevant to the long-term goal of this study. In radiology, both raw images and RRs are stored electronically in picture archiving and communication systems (PACS) and electronic medical records (EMR) and thus, gathering a large number of images is theoretically feasible. However, raw MRIs, with their large volume of voxel information (>10^6^) and inconsistent positions and orientations, are unsuitable for direct large-scale analyses. Analyzing available RRs in free-text formats would be even more problematic, because the six error categories described above for AS generation apply to human-generated sentences. For example, the names given to specific anatomic locations are not well defined or consistent among radiologists. Depending on the educational background of a radiologist, s/he might use different anatomic names to indicate similar locations. Furthermore, some structures (e.g., ventricles, amygdala) might be frequently evaluated and recorded by one radiologist, but not by others. In addition, threshold values and degree of age compensation used when judging abnormality are inconsistent among radiologists. A term such as “moderate atrophy” might mean either normal for that patient's age or modest abnormality. Ingesting raw data from PACS and EMR would cause considerable difficulties. Obviously, some form of metadata sitting between raw images and free-text RRs are needed to ingest a large amount of radiologic data.

## Conclusions

This paper describes our initial attempts to automatically generate RRs based on quantitative brain segmentation tools. The translation from raw images with >10^6^ voxels to a few clinically meaningful sentences involved a series of information-reduction and -conversion processes, which raised several important questions about the future potential of such tools as well as ambiguity in the thought processes of radiologists. The most significant discrepancy between the human and automatically generated RRs was caused by the sentence-triggering threshold (the degree of abnormality), which was fixed to z-score >2.0 for the automated generation, while the thresholds by radiologists varied among different anatomical structures. This type of computer-aided approach might play an important role in more evidence-based image reading in the future.

## Author Contributions

KA, RS, KO, AF, and SM contributed to the design of the study. CL collected data. YL, MM, and SM developed tools for data processing. DW and YL performed the database preprocessing. KA, RS, and SN analyzed the data. KA and SM interpreted the results. KA and SM performed the drafting of the article. KA, KY, KT, CL, and SM reviewed of the entire manuscript. All authors approved the manuscript.

### Conflict of Interest Statement

SM and MM own “AnatomyWorks.” SM is its CEO. This arrangement is being managed by the Johns Hopkins University in accordance with its conflict-of-interest policies. The remaining authors declare that the research was conducted in the absence of any commercial or financial relationships that could be construed as a potential conflict of interest.

## Abbreviations

RR: radiologic report
MR: magnetic resonance
MPRAGE: magnetization-prepared rapid gradient-echo
ADNI: Alzheimer's Disease Neuroimaging Initiative
SD: standard deviation
AS: automated sentence
TP: true-positive
FN: false-negative
FP: false-positive
TN: true-negative
PACS: picture archiving and communication systems
EMR: electronic medical records.
