# Supplementary material for: Automated Generation of Radiologic Descriptions on Brain Volume Changes From T1-Weighted MR Images: Initial Assessment of Feasibility
Source: Front Neurol. 2019 Jan 24;10:7. doi: 10.3389/fneur.2019.00007 (PMC6354548; doi:10.3389/fneur.2019.00007)
Supplement: Supplementary file 1 [file Table_1.DOCX]

**Automated Generation of Radiologic Descriptions on Brain Volume Changes from T1-weighted MR Images**

Kentaro Akazawa, Ryo Sakamoto, Satoshi Nakajima, Dan Wu, Yue Li, Kenichi Oishi, Andreia V. Faria, Kei Yamada, Kaori Togashi, Constantine G. Lyketsos, Michael I. Miller, Susumu Mori *; For the Alzheimer’s Disease Neuroimaging Initiative

*Correspondence: Dr. Susumu Mori: [smori1@jhmi.edu](mailto:smori1@jhmi.edu)

Supplementary Table 1: Preset clinical knowledge filer

| Ontology Level | Level 1 | | Level 3 | | Level 5 | |
| --- | --- | --- | --- | --- | --- | --- |
| Brain tissue | Rt | Hemisphere | Rt | Frontal lobe | Rt | Caudate nucleus |
|  |  |  | Lt |  | Lt |  |
|  |  |  | Rt | Parietal lobe |  |  |
|  |  |  | Lt |  |  |  |
|  |  |  | Rt | Temporal lobe |  |  |
|  | Lt |  | Lt |  |  |  |
|  |  |  | Rt | Occipital lobe |  |  |
|  |  |  | Lt |  |  |  |
|  |  |  | Rt | Limbic system | Rt | Hippocampus |
|  |  |  | Lt |  | Lt |  |
| Ventricle |  |  | Rt | Lateral ventricle |  |  |
|  |  |  | Lt |  |  |  |
|  |  |  |  | Third ventricle |  |  |
|  |  |  |  | Forth ventricle |  |  |

Lt: left, Rt: right
