# Supplementary material for: Automated Generation of Radiologic Descriptions on Brain Volume Changes From T1-Weighted MR Images: Initial Assessment of Feasibility
Source: Front Neurol. 2019 Jan 24;10:7. doi: 10.3389/fneur.2019.00007 (PMC6354548; doi:10.3389/fneur.2019.00007)
Supplement: Supplementary file 2 [file Table_2.DOCX]

**Automated Generation of Radiologic Descriptions on Brain Volume Changes from T1-weighted MR Images**

Kentaro Akazawa, Ryo Sakamoto, Satoshi Nakajima, Dan Wu, Yue Li, Kenichi Oishi, Andreia V. Faria, Kei Yamada, Kaori Togashi, Constantine G. Lyketsos, Michael I. Miller, Susumu Mori *; For the Alzheimer’s Disease Neuroimaging Initiative

*Correspondence: Dr. Susumu Mori: [smori1@jhmi.edu](mailto:smori1@jhmi.edu)

Supplementary Table 2: The Boolean operations

| AS | Tier | ROI #1 |  | ROI #2 |  | ROI #3 |  | ROI #4 |  | ROI #5 |  | ROI #6 |  | ROI #7 |  | ROI #8 |  | ROI #9 |  | ROI #10 |  | ROI #11 |  | ROI #12 |
| --- | --- | --- | --- | --- | --- | --- | --- | --- | --- | --- | --- | --- | --- | --- | --- | --- | --- | --- | --- | --- | --- | --- | --- | --- |
| There is bilateral He atrophy | 1 | "L1_He_L"< -2 | & | "L1_He_R"< -2 |  |  |  |  |  |  |  |  |  |  |  |  |  |  |  |  |  |  |  |  |
| The atrophy is prominent in the Fr lobe | 2 | "L3_Fr_L"< -2 | & | "L3_Fr_R"< -2 | & | "L3_Pa_L"> -2 | & | "L3_Pa_R"> -2 | & | "L3_Te_L"> -2 | & | "L3_Te_R"> -2 | & | "L3_Oc_L"> -2 | & | "L3_Oc_R"> -2 | & | "L3_Li_L"> -2 | & | "L3_Li_R"> -2 |  |  |  |  |
| The atrophy is prominent in the Pa lobe | 2 | "L3_Fr_L"> -2 | & | "L3_Fr_R"> -2 | & | "L3_Pa_L"< -2 | & | "L3_Pa_R"< -2 | & | "L3_Te_L"> -2 | & | "L3_Te_R"> -2 | & | "L3_Oc_L"> -2 | & | "L3_Oc_R"> -2 | & | "L3_Li_L"> -2 | & | "L3_Li_R"> -2 |  |  |  |  |
| The atrophy is prominent in the Te lobe | 2 | "L3_Fr_L"> -2 | & | "L3_Fr_R"> -2 | & | "L3_Pa_L"> -2 | & | "L3_Pa_R"> -2 | & | "L3_Te_L"< -2 | & | "L3_Te_R"< -2 | & | "L3_Oc_L"> -2 | & | "L3_Oc_R"> -2 | & | "L3_Li_L"> -2 | & | "L3_Li_R"> -2 |  |  |  |  |
| The atrophy is prominent in the Oc lobe | 2 | "L3_Fr_L"> -2 | & | "L3_Fr_R"> -2 | & | "L3_Pa_L"> -2 | & | "L3_Pa_R"> -2 | & | "L3_Te_L"> -2 | & | "L3_Te_R"> -2 | & | "L3_Oc_L"< -2 | & | "L3_Oc_R"< -2 | & | "L3_Li_L"> -2 | & | "L3_Li_R"> -2 |  |  |  |  |
| The atrophy is prominent in the Li lobe | 2 | "L3_Fr_L"> -2 | & | "L3_Fr_R"> -2 | & | "L3_Pa_L"> -2 | & | "L3_Pa_R"> -2 | & | "L3_Te_L"> -2 | & | "L3_Te_R"> -2 | & | "L3_Oc_L"> -2 | & | "L3_Oc_R"> -2 | & | "L3_Li_L"< -2 | & | "L3_Li_R"< -2 |  |  |  |  |
| The atrophy is prominent in the Fr and Te lobe | 2 | "L3_Fr_L"< -2 | & | "L3_Fr_R"< -2 | & | "L3_Pa_L"> -2 | & | "L3_Pa_R"> -2 | & | "L3_Te_L"< -2 | & | "L3_Te_R"< -2 | & | "L3_Oc_L"> -2 | & | "L3_Oc_R"> -2 | & | "L3_Li_L"> -2 | & | "L3_Li_R"> -2 |  |  |  |  |
| The atrophy is prominent in the Fr and Pa lobe | 2 | "L3_Fr_L"< -2 | & | "L3_Fr_R"< -2 | & | "L3_Pa_L"< -2 | & | "L3_Pa_R"< -2 | & | "L3_Te_L"> -2 | & | "L3_Te_R"> -2 | & | "L3_Oc_L"> -2 | & | "L3_Oc_R"> -2 | & | "L3_Li_L"> -2 | & | "L3_Li_R"> -2 |  |  |  |  |
| There is L He atrophy | 1 | "L1_He_L"< -2 | & | "L1_He_R"> -2 |  |  |  |  |  |  |  |  |  |  |  |  |  |  |  |  |  |  |  |  |
| The atrophy is prominent in the Fr lobe | 2 | "L3_Fr_L"< -2 |  |  | & | "L3_Pa_L"> -2 |  |  | & | "L3_Te_L"> -2 |  |  | & | "L3_Oc_L"> -2 |  |  | & | "L3_Li_L"> -2 |  |  |  |  |  |  |
| The atrophy is prominent in the Pa lobe | 2 | "L3_Fr_L"> -2 |  |  | & | "L3_Pa_L"< -2 |  |  | & | "L3_Te_L"> -2 |  |  | & | "L3_Oc_L"> -2 |  |  | & | "L3_Li_L"> -2 |  |  |  |  |  |  |
| The atrophy is prominent in the Te lobe | 2 | "L3_Fr_L"> -2 |  |  | & | "L3_Pa_L"> -2 |  |  | & | "L3_Te_L"< -2 |  |  | & | "L3_Oc_L"> -2 |  |  | & | "L3_Li_L"> -2 |  |  |  |  |  |  |
| The atrophy is prominent in the Oc lobe | 2 | "L3_Fr_L"> -2 |  |  | & | "L3_Pa_L"> -2 |  |  | & | "L3_Te_L"> -2 |  |  | & | "L3_Oc_L"< -2 |  |  | & | "L3_Li_L"> -2 |  |  |  |  |  |  |
| The atrophy is prominent in the Li lobe | 2 | "L3_Fr_L"> -2 |  |  | & | "L3_Pa_L"> -2 |  |  | & | "L3_Te_L"> -2 |  |  | & | "L3_Oc_L"> -2 |  |  | & | "L3_Li_L"< -2 |  |  |  |  |  |  |
| The atrophy is prominent in the Fr and Te lobe | 2 | "L3_Fr_L"< -2 |  |  | & | "L3_Pa_L"> -2 |  |  | & | "L3_Te_L"< -2 |  |  | & | "L3_Oc_L"> -2 |  |  | & | "L3_Li_L"> -2 |  |  |  |  |  |  |
| The atrophy is prominent in the Fr and Pa lobe | 2 | "L3_Fr_L"< -2 |  |  | & | "L3_Pa_L"< -2 |  |  | & | "L3_Te_L"> -2 |  |  | & | "L3_Oc_L"> -2 |  |  | & | "L3_Li_L"> -2 |  |  |  |  |  |  |
| There is R He atrophy | 1 | "L1_He_L"> -2 | & | "L1_He_R"< -2 |  |  |  |  |  |  |  |  |  |  |  |  |  |  |  |  |  |  |  |  |
| The atrophy is prominent in the Fr lobe | 2 |  |  | "L3_Fr_R"< -2 | & |  |  | "L3_Pa_R"> -2 | & |  |  | "L3_Te_R"> -2 | & |  |  | "L3_Oc_R"> -2 | & |  |  | "L3_Li_R"> -2 |  |  |  |  |
| The atrophy is prominent in the Pa lobe | 2 |  |  | "L3_Fr_R"> -2 | & |  |  | "L3_Pa_R"< -2 | & |  |  | "L3_Te_R"> -2 | & |  |  | "L3_Oc_R"> -2 | & |  |  | "L3_Li_R"> -2 |  |  |  |  |
| The atrophy is prominent in the Te lobe | 2 |  |  | "L3_Fr_R"> -2 | & |  |  | "L3_Pa_R"> -2 | & |  |  | "L3_Te_R"< -2 | & |  |  | "L3_Oc_R"> -2 | & |  |  | "L3_Li_R"> -2 |  |  |  |  |
| The atrophy is prominent in the Oc lobe | 2 |  |  | "L3_Fr_R"> -2 | & |  |  | "L3_Pa_R"> -2 | & |  |  | "L3_Te_R"> -2 | & |  |  | "L3_Oc_R"< -2 | & |  |  | "L3_Li_R"> -2 |  |  |  |  |
| The atrophy is prominent in the Li lobe | 2 |  |  | "L3_Fr_R"> -2 | & |  |  | "L3_Pa_R"> -2 | & |  |  | "L3_Te_R"> -2 | & |  |  | "L3_Oc_R"> -2 | & |  |  | "L3_Li_R"< -2 |  |  |  |  |
| The atrophy is prominent in the Fr and Te lobe | 2 |  |  | "L3_Fr_R"< -2 | & |  |  | "L3_Pa_R"> -2 | & |  |  | "L3_Te_R"< -2 | & |  |  | "L3_Oc_R"> -2 | & |  |  | "L3_Li_R"> -2 |  |  |  |  |
| The atrophy is prominent in the Fr and Pa lobe | 2 |  |  | "L3_Fr_R"< -2 | & |  |  | "L3_Pa_R"< -2 | & |  |  | "L3_Te_R"> -2 | & |  |  | "L3_Oc_R"> -2 | & |  |  | "L3_Li_R"> -2 |  |  |  |  |
| There is bi-He Fr lobe specific atrophy | 1 | "L1_He_L"> -2 | & | "L1_He_R"> -2 | & | "L3_Fr_L"< -2 | & | "L3_Fr_R"< -2 | & | "L3_Pa_L"> -2 | & | "L3_Pa_R"> -2 | & | "L3_Te_L"> -2 | & | "L3_Te_R"> -2 | & | "L3_Oc_L"> -2 | & | "L3_Oc_R"> -2 | & | "L3_Li_L"> -2 | & | "L3_Li_R"> -2 |
| There is L Fr lobe specific atrophy | 1 | "L1_He_L"> -2 | & | "L1_He_R"> -2 | & | "L3_Fr_L"< -2 | & | "L3_Fr_R"> -2 | & | "L3_Pa_L"> -2 | & | "L3_Pa_R"> -2 | & | "L3_Te_L"> -2 | & | "L3_Te_R"> -2 | & | "L3_Oc_L"> -2 | & | "L3_Oc_R"> -2 | & | "L3_Li_L"> -2 | & | "L3_Li_R"> -2 |
| There is R Fr lobe specific atrophy | 1 | "L1_He_L"> -2 | & | "L1_He_R"> -2 | & | "L3_Fr_L"> -2 | & | "L3_Fr_R"< -2 | & | "L3_Pa_L"> -2 | & | "L3_Pa_R"> -2 | & | "L3_Te_L"> -2 | & | "L3_Te_R"> -2 | & | "L3_Oc_L"> -2 | & | "L3_Oc_R"> -2 | & | "L3_Li_L"> -2 | & | "L3_Li_R"> -2 |
| There is bi-He Pa lobe specific atrophy | 1 | "L1_He_L"> -2 | & | "L1_He_R"> -2 | & | "L3_Fr_L"> -2 | & | "L3_Fr_R"> -2 | & | "L3_Pa_L"< -2 | & | "L3_Pa_R"< -2 | & | "L3_Te_L"> -2 | & | "L3_Te_R"> -2 | & | "L3_Oc_L"> -2 | & | "L3_Oc_R"> -2 | & | "L3_Li_L"> -2 | & | "L3_Li_R"> -2 |
| There is L Pa lobe specific atrophy | 1 | "L1_He_L"> -2 | & | "L1_He_R"> -2 | & | "L3_Fr_L"> -2 | & | "L3_Fr_R"> -2 | & | "L3_Pa_L"< -2 | & | "L3_Pa_R"> -2 | & | "L3_Te_L"> -2 | & | "L3_Te_R"> -2 | & | "L3_Oc_L"> -2 | & | "L3_Oc_R"> -2 | & | "L3_Li_L"> -2 | & | "L3_Li_R"> -2 |
| There is R Pa lobe specific atrophy | 1 | "L1_He_L"> -2 | & | "L1_He_R"> -2 | & | "L3_Fr_L"> -2 | & | "L3_Fr_R"> -2 | & | "L3_Pa_L"> -2 | & | "L3_Pa_R"< -2 | & | "L3_Te_L"> -2 | & | "L3_Te_R"> -2 | & | "L3_Oc_L"> -2 | & | "L3_Oc_R"> -2 | & | "L3_Li_L"> -2 | & | "L3_Li_R"> -2 |
| There is bi-He Te lobe specific atrophy | 1 | "L1_He_L"> -2 | & | "L1_He_R"> -2 | & | "L3_Fr_L"> -2 | & | "L3_Fr_R"> -2 | & | "L3_Pa_L"> -2 | & | "L3_Pa_R"> -2 | & | "L3_Te_L"< -2 | & | "L3_Te_R"< -2 | & | "L3_Oc_L"> -2 | & | "L3_Oc_R"> -2 | & | "L3_Li_L"> -2 | & | "L3_Li_R"> -2 |
| There is L Te lobe specific atrophy | 1 | "L1_He_L"> -2 | & | "L1_He_R"> -2 | & | "L3_Fr_L"> -2 | & | "L3_Fr_R"> -2 | & | "L3_Pa_L"> -2 | & | "L3_Pa_R"> -2 | & | "L3_Te_L"< -2 | & | "L3_Te_R"> -2 | & | "L3_Oc_L"> -2 | & | "L3_Oc_R"> -2 | & | "L3_Li_L"> -2 | & | "L3_Li_R"> -2 |
| There is R Te lobe specific atrophy | 1 | "L1_He_L"> -2 | & | "L1_He_R"> -2 | & | "L3_Fr_L"> -2 | & | "L3_Fr_R"> -2 | & | "L3_Pa_L"> -2 | & | "L3_Pa_R"> -2 | & | "L3_Te_L"> -2 | & | "L3_Te_R"< -2 | & | "L3_Oc_L"> -2 | & | "L3_Oc_R"> -2 | & | "L3_Li_L"> -2 | & | "L3_Li_R"> -2 |
| There is bi-He Oc lobe specific atrophy | 1 | "L1_He_L"> -2 | & | "L1_He_R"> -2 | & | "L3_Fr_L"> -2 | & | "L3_Fr_R"> -2 | & | "L3_Pa_L"> -2 | & | "L3_Pa_R"> -2 | & | "L3_Te_L"> -2 | & | "L3_Te_R"> -2 | & | "L3_Oc_L"< -2 | & | "L3_Oc_R"< -2 | & | "L3_Li_L"> -2 | & | "L3_Li_R"> -2 |
| There is L Oc lobe specific atrophy | 1 | "L1_He_L"> -2 | & | "L1_He_R"> -2 | & | "L3_Fr_L"> -2 | & | "L3_Fr_R"> -2 | & | "L3_Pa_L"> -2 | & | "L3_Pa_R"> -2 | & | "L3_Te_L"> -2 | & | "L3_Te_R"> -2 | & | "L3_Oc_L"< -2 | & | "L3_Oc_R"> -2 | & | "L3_Li_L"> -2 | & | "L3_Li_R"> -2 |
| There is R Oc lobe specific atrophy | 1 | "L1_He_L"> -2 | & | "L1_He_R"> -2 | & | "L3_Fr_L"> -2 | & | "L3_Fr_R"> -2 | & | "L3_Pa_L"> -2 | & | "L3_Pa_R"> -2 | & | "L3_Te_L"> -2 | & | "L3_Te_R"> -2 | & | "L3_Oc_L"> -2 | & | "L3_Oc_R"< -2 | & | "L3_Li_L"> -2 | & | "L3_Li_R"> -2 |
| There is bi-He Li specific atrophy | 1 | "L1_He_L"> -2 | & | "L1_He_R"> -2 | & | "L3_Fr_L"> -2 | & | "L3_Fr_R"> -2 | & | "L3_Pa_L"> -2 | & | "L3_Pa_R"> -2 | & | "L3_Te_L"> -2 | & | "L3_Te_R"> -2 | & | "L3_Oc_L"> -2 | & | "L3_Oc_R"> -2 | & | "L3_Li_L"< -2 | & | "L3_Li_R"< -2 |
| There is L Li specific atrophy | 1 | "L1_He_L"> -2 | & | "L1_He_R"> -2 | & | "L3_Fr_L"> -2 | & | "L3_Fr_R"> -2 | & | "L3_Pa_L"> -2 | & | "L3_Pa_R"> -2 | & | "L3_Te_L"> -2 | & | "L3_Te_R"> -2 | & | "L3_Oc_L"> -2 | & | "L3_Oc_R"> -2 | & | "L3_Li_L"< -2 | & | "L3_Li_R"> -2 |
| There is R Li specific atrophy | 1 | "L1_He_L"> -2 | & | "L1_He_R"> -2 | & | "L3_Fr_L"> -2 | & | "L3_Fr_R"> -2 | & | "L3_Pa_L"> -2 | & | "L3_Pa_R"> -2 | & | "L3_Te_L"> -2 | & | "L3_Te_R"> -2 | & | "L3_Oc_L"> -2 | & | "L3_Oc_R"> -2 | & | "L3_Li_L"> -2 | & | "L3_Li_R"< -2 |
| There is bi-He Fr-Te specific atrophy | 1 | "L1_He_L"> -2 | & | "L1_He_R"> -2 | & | "L3_Fr_L"< -2 | & | "L3_Fr_R"< -2 | & | "L3_Pa_L"> -2 | & | "L3_Pa_R"> -2 | & | "L3_Te_L"< -2 | & | "L3_Te_R"< -2 | & | "L3_Oc_L"> -2 | & | "L3_Oc_R"> -2 | & | "L3_Li_L"> -2 | & | "L3_Li_R"> -2 |
| There is L Fr-Te specific atrophy | 1 | "L1_He_L"> -2 | & | "L1_He_R"> -2 | & | "L3_Fr_L"< -2 | & | "L3_Fr_R"> -2 | & | "L3_Pa_L"> -2 | & | "L3_Pa_R"> -2 | & | "L3_Te_L"< -2 | & | "L3_Te_R"> -2 | & | "L3_Oc_L"> -2 | & | "L3_Oc_R"> -2 | & | "L3_Li_L"> -2 | & | "L3_Li_R"> -2 |
| There is R Fr-Te specific atrophy | 1 | "L1_He_L"> -2 | & | "L1_He_R"> -2 | & | "L3_Fr_L"> -2 | & | "L3_Fr_R"< -2 | & | "L3_Pa_L"> -2 | & | "L3_Pa_R"> -2 | & | "L3_Te_L"> -2 | & | "L3_Te_R"< -2 | & | "L3_Oc_L"> -2 | & | "L3_Oc_R"> -2 | & | "L3_Li_L"> -2 | & | "L3_Li_R"> -2 |
| There is bi-He Fr-Pa specific atrophy | 1 | "L1_He_L"> -2 | & | "L1_He_R"> -2 | & | "L3_Fr_L"< -2 | & | "L3_Fr_R"< -2 | & | "L3_Pa_L"< -2 | & | "L3_Pa_R"< -2 | & | "L3_Te_L"> -2 | & | "L3_Te_R"> -2 | & | "L3_Oc_L"> -2 | & | "L3_Oc_R"> -2 | & | "L3_Li_L"> -2 | & | "L3_Li_R"> -2 |
| There is L Fr-Pa specific atrophy | 1 | "L1_He_L"> -2 | & | "L1_He_R"> -2 | & | "L3_Fr_L"< -2 | & | "L3_Fr_R"> -2 | & | "L3_Pa_L"< -2 | & | "L3_Pa_R"> -2 | & | "L3_Te_L"> -2 | & | "L3_Te_R"> -2 | & | "L3_Oc_L"> -2 | & | "L3_Oc_R"> -2 | & | "L3_Li_L"> -2 | & | "L3_Li_R"> -2 |
| There is R Fr-Pa specific atrophy | 1 | "L1_He_L"> -2 | & | "L1_He_R"> -2 | & | "L3_Fr_L"> -2 | & | "L3_Fr_R"< -2 | & | "L3_Pa_L"> -2 | & | "L3_Pa_R"< -2 | & | "L3_Te_L"> -2 | & | "L3_Te_R"> -2 | & | "L3_Oc_L"> -2 | & | "L3_Oc_R"> -2 | & | "L3_Li_L"> -2 | & | "L3_Li_R"> -2 |
| The Hi has bi-lateral atrophy | 1 | "L5_Hi_L"< -2 | & | "L5_Hi_R"< -2 |  |  |  |  |  |  |  |  |  |  |  |  |  |  |  |  |  |  |  |  |
| The L Hi has atrophy | 1 | "L5_Hi_L"< -2 | & | "L5_Hi_R"> -2 |  |  |  |  |  |  |  |  |  |  |  |  |  |  |  |  |  |  |  |  |
| The R Hi has atrophy | 1 | "L5_Hi_L"> -2 | & | "L5_Hi_R"< -2 |  |  |  |  |  |  |  |  |  |  |  |  |  |  |  |  |  |  |  |  |
| The caudate has bi-lateral atrophy | 1 | "L5_Caud_L"< -2 | & | "L5_Caud_R"< -2 |  |  |  |  |  |  |  |  |  |  |  |  |  |  |  |  |  |  |  |  |
| The L caudate has atrophy | 1 | "L5_Caud_L"< -2 | & | "L5_Caud_R"> -2 |  |  |  |  |  |  |  |  |  |  |  |  |  |  |  |  |  |  |  |  |
| The R caudate has atrophy | 1 | "L5_Caud_L"> -2 | & | "L5_Caud_R"< -2 |  |  |  |  |  |  |  |  |  |  |  |  |  |  |  |  |  |  |  |  |
|  |  |  |  |  |  |  |  |  |  |  |  |  |  |  |  |  |  |  |  |  |  |  |  |  |
| There is global enlargement of the LVs | 1 | "L3_LV_L"> 2 | & | "L3_LV_R"> 2 |  |  |  |  |  |  |  |  |  |  |  |  |  |  |  |  |  |  |  |  |
| There is enlargement of the L LVs | 1 | "L3_LV_L"> 2 | & | "L3_LV_R"< 2 |  |  |  |  |  |  |  |  |  |  |  |  |  |  |  |  |  |  |  |  |
| There is enlargement of the R LVs | 1 | "L3_LV_L"< 2 | & | "L3_LV_R"> 2 |  |  |  |  |  |  |  |  |  |  |  |  |  |  |  |  |  |  |  |  |
| The III is enlarged | 1 | "L3_III_V"> 2 | & |  |  |  |  |  |  |  |  |  |  |  |  |  |  |  |  |  |  |  |  |  |
| The IV is enlarged | 1 | "L3_IV_V"> 2 | & |  |  |  |  |  |  |  |  |  |  |  |  |  |  |  |  |  |  |  |  |  |

AS: Automated sentence, Ca; caudate nucleus, Fr: frontal, He: hemispheric, Hi: hippocampus, III: third ventricle, IV: forth ventricle, L: left, L1-5: level 1-5, Li: limbic, LV: lateral ventricle, Oc: occipital, Pa: parietal, R = right, Te: temporal
